# Fear Factor in Seeking Dental Care among Saudis during COVID-19 Pandemic

**DOI:** 10.3390/ijerph182010589

**Published:** 2021-10-09

**Authors:** Maria Salem Ibrahim, Hala Alibrahim, Abdullah Al Madani, Abdulaziz Alamri, Mohamed Bamashmous, Abrar Tounsi

**Affiliations:** 1 Department of Preventive Dental Sciences, College of Dentistry, Imam Abdulrahman bin Faisal University, Dammam 34212, Saudi Arabia; absalamri@iau.edu.sa; 2 College of Dentistry, Imam Abdulrahman Bin Faisal University, Dammam 34212, Saudi Arabia; 2150003599@iau.edu.sa; 3 Dental Hospital, College of Dentistry, Imam Abdulrahman bin Faisal University, Dammam 34212, Saudi Arabia; aaalmadani@iau.edu.sa; 4 Department of Dental Public Health, Faculty of Dentistry, King Abdulaziz University, Jeddah 21589, Saudi Arabia; Mbamashmous@kau.edu.sa; 5 Department of Periodontics and Community Dentistry, College of Dentistry, King Saud University, Riyadh 11451, Saudi Arabia

**Keywords:** dental care, fear, COVID-19, oral health

## Abstract

The recent coronavirus disease of 2019 (COVID-19) pandemic led to major lifestyle changes. The present study sought to assess factors associated with fear to seek dental care during COVID-19 pandemic in Saudi Arabia. This cross-sectional study was conducted during the COVID-19 outbreak in 2020. An online questionnaire was filled by a convenient sample of adult Saudi residents through mobile instant messaging application. The following measures were collected: sociodemographic characteristics, fear of COVID-19 using validated Fears of Illness and Virus Evaluation scale, fear to seek dental care, perceived health status, and COVID-19 experience. There were 826 participants involved in this study (541 females and 285 males, mean age: 38.8 ± 13.29 years). Fear to seek dental care was significantly higher among females, younger age groups, people who perceived poor general and oral health, and people who perceived high risk of contracting the virus in dental clinics. After controlling for confounders, fear to seek dental care was significantly higher among the age group of 35–44 years, those who perceived high and moderate risk of COVID-19 infection in dental clinics, and among participants who reported untreated dental conditions. Fear that Others Get Sick, Fear of the Impact on Social Life, and Behaviors Related to Illness and Virus Fears were significantly associated with high levels of fear to seek dental care. Within the study’s limitations, fear of COVID-19 negatively impacted the study population’s willingness to seek dental treatment. Factors such as age, perceived risk of COVID-19 infection in dental clinics, and untreated dental conditions were associated with fear to seek dental care.

## 1. Introduction

Humans encounter multiple threats to their health and safety, such as bioterrorism and newly emerging infectious diseases. On 30 January 2020, coronavirus disease of 2019 (COVID-19) has been characterized by the World Health Organization (WHO) as a public health emergency of an international concern [1]. By July 2021, over 190 million COVID-19 cases and over 4 million deaths were registered worldwide [2]. Therefore, the need for medical supplies and units increased [3]. As response to this critical situation, many countries placed travel restrictions and quarantine to control the spread of the infection [4]. In addition, the WHO provided infection control measures to limit the spread of COVID-19, such as social distancing through avoiding closeness to others, mandating face masks, and avoiding contact with wild animals [5]. The effect of COVID-19 outbreak on various sectors negatively influenced people worldwide. A crucial aspect during this pandemic is considering individuals’ fears among all these stressors for their psychological wellbeing [6,7].

With the global spread of this virus, a considerable degree of fear was spreading among the public [7,8]. Social networks during the pandemic generated various alarming news about the infection among the population [9]. The associated COVID-19 fear poses a psychological burden expressed as anxiety, stress, and depression [10]. Fear is a common defensive emotion that can be inherited or learned [11]. It consists of a series of biological steps designed to prepare humans for an intimidating event [11]. Fear is a multidimensional construct that could be reflected by a conglomerate of physiological, behavioral, and subjective responses [12].

Wang et al. (2020) reported that 53.8% of the Chinese population had a moderate to severe negative psychological impact in regard to COVID-19, 28.8% had anxiety disorders, 16.5% had depressive symptoms, and 8.1% expressed moderate to severe levels of stress [13]. COVID-19 outbreak was associated with higher levels of fear, anxiety and depression among adults in Saudi Arabia [14,15]. Alkhamees et al. (2020) reported that 23.6% of the general population of Saudi Arabia had a moderate or severe psychological impact [15]. In the aforementioned study, severe symptoms of stress were expressed by 13.7%, 13.9% experienced severe symptoms of anxiety, and 16.4% experienced severe symptoms of depression [15]. Furthermore, lockdowns affected our lifestyle and resulted in various levels of negative psychological impacts [16,17]. In a study conducted among the Chinese population, people who lived in quarantined areas expressed increased fear of getting infected and needed more psychological support than those in non-quarantined areas [18]. These psychological disturbances, including fear, can influence health and healthcare-seeking behaviors.

Oral health is an integral part of general health. Many oral diseases are associated with systemic diseases, such as cardiovascular diseases, diabetes, etc. [19,20]. Dental conditions, such as dental caries are highly prevalent among the Saudi population [21,22]. Dental diseases, if left untreated, can impede the quality of life of the affected individuals. Not seeking dental services may delay the diagnosis, and result in problematic untreated oral conditions. Dental care is critical to maintain a good dental health and prevent the harmful effect of oral diseases on the quality of life [23].

As the transmission of COVID-19 is exponential and dental clinics are considered as a high-risk environment for virus transmission [24], we can predict an increased level of fear from visiting dental clinics and getting dental treatment during this pandemic. Adverse health outcomes can be expected because of the fear from such outbreaks, which may affect the accessibility and willingness of seeking treatment. Another factor associated with the healthcare seeking behavior is the level of confidence in the provided service [23,25].

Failure to seek dental care can negatively impact oral health status. Even though some studies looked into the barriers to dental visits during the pandemic in Saudi Arabia [26,27,28], factors related to higher fear and reluctance to seek dental care were not widely investigated. Considering these factors is essential for targeting these specific populations in preventive dental programs during the current and future pandemics. Hence, this study aimed to assess fear among residents of Saudi Arabia to seek dental care during the pandemic and the factors associated with it. 

## 2. Materials and Methods

This cross-sectional study was approved by King Saud University Institutional Review Board (E-20-4792). The target population was adults aged 18 years and older who reside in Saudi Arabia. Data were collected using an online-based self-administered survey between May and June 2020. The calculated minimum sample size was 385 using the 2019 Saudi population size (34.27 million), 5% margin of error (MOE), 95% confidence level and 50% sample proportion. Due to the difficulty of collecting data during COVID-19 lockdown, a non-probability convenient sampling and snowballing techniques were used to recruit participants via WhatsApp. By submitting the form, participants consented to participate in the study. Confidentiality was assured by collecting non-identifying data. 

### 2.1. Measures

The survey consisted of the following sections: sociodemographic characteristics (gender, age, citizenship, region, monthly income, education, and working in the medical field), Fear of Illness and Virus Evaluation (FIVE), fear to seek dental care, dental services utilization, risk of COVID-19 infection in dental clinics, perception of health, and COVID-19 experience. The content of an Arabic version of the FIVE was validated in another study [29], then the subscales were reconstructed accordingly. The survey was pilot tested on selected individuals from the same target population.

#### 2.1.1. Fear of Illness and Virus Evaluation (FIVE; Ehrenreich-May)

Fear of COVID-19 was the independent variable, which was measured using the FIVE scale. The FIVE has three versions (adult, child, and parent). The adult reconstructed version [29] consists of four subscales (Fear of Getting Sick (4 items), Fear that Others Get Sick (4 items), Fear of the Impact on Social Life (13 items) and Behaviors Related to Illness, and Virus Fears (14 items)) with 35 items in total. In the first three subscales, participants were asked to rate the frequency of feeling fear during the last week on a Likert scale of four (1 for “I am not afraid of this at all”, 2 for “ I am afraid of this some of the time”, 3 for “ I am afraid of this most of the time”, and 4 for “I am afraid of this all of the time”), and the truthfulness of two statements on a Likert scale of four (1 for “not true for me at all”, 2 for “somewhat true”, 3 for “mostly true”, and 4 for “definitely true”). The fourth subscale includes rating the frequency of doing specific behaviors within the last week on a Likert scale of four (1 for “I have not done this in last week” and 4 for “I did this all the time last week”).

In the present study, some modifications were made on the FIVE validated version [29] to accommodate for the time of data collection and the sample population. Two of the items in Fear of the Impact on Social Life subscale that were related to pets and attending to work were omitted in this version of the survey. This was due to the rareness of owning pets among the Saudi population, and the lockdown that could prevent the individuals from attending their work. This makes the items in Fear of the Impact on Social Life subscale 11 items. Another two items in Behaviors Related to Illness and Virus Fears subscale, which were related to working using the computer and practicing sports outdoors, were omitted in this version. The omission of these two items was because not everyone has work to be done on a computer, and the lockdown that could prevent people from practicing sports outdoors. This makes the items in Behaviors Related to Illness and Virus Fears subscale 12 items.

The sum score for each subscale in the modified version of FIVE in the present study has changed where the maximum sum score for Fears of Getting Sick subscale is 16, for Fears that Others Get Sick subscale is 16, for Fear of the Impact on Social Life is 44, and Behaviors Related to Illness and Virus Fears subscale is 48. 

#### 2.1.2. Fear to Seek Dental Care 

Reluctance to seek dental care due to fear was the dependent variable, which was assessed using two items (“my fears from coronavirus infection prevent me from visiting the dentist for emergency”, “my fears from coronavirus infection get in the way of trusting infection control measures taken in dental clinics”). The respondents were asked to rate the truthfulness of each statement on a Likert scale of four (1 for “not true for me at all”, 2 for “somewhat true”, 3 for “mostly true”, and 4 for “definitely true”). As seeking healthcare was linked to fear and confidence in the level of provided care [25,27], these two items were developed for the purpose of the present study following FIVE format. A group of four dental public health experts approved the appropriateness of the items in estimating fear to seek dental care during the pandemic. The clarity and appropriateness of the questions were further pilot tested on a sample of 15 individuals. The mean of Fear to seek dental care was calculated by dividing the sum score of the two items by 2. 

#### 2.1.3. Dental Services Utilization

It was determined by the frequencies of dental visits, seeing a dentist within the last 6 months, having a regular dentist [30], and reporting untreated dental conditions. The frequency of the dental visits was measured by the question “How often do you see a dental professional?” and answers included “more than once a year, about once a year, only for emergency care, never”. Untreated dental conditions were estimated by asking participants “Do you think you have any untreated conditions in your mouth?”

#### 2.1.4. Perceived Risk of COVID-19 Infection in Dental Clinics 

Participants were requested to estimate the perceived risk of contracting coronavirus when attending a dental clinic on a scale of four (1 for “no risk”, 2 for “low risk”, 3 for “moderate risk”, and 4 for “high risk”). 

#### 2.1.5. Perception of Health

It was assessed by requesting the participants to rate their own general health and oral health. Rating was on a scale of four (1 for “excellent”, 2 for “good”, 3 for “fair”, and 4 for “poor”). Participants were further asked to estimate the importance of oral health on a scale of four (1 for “very important”, 2 for “somewhat important”, 3 for “not important”, and 4 for “not important at all”). 

#### 2.1.6. COVID-19 Experience 

Participants’ experience with COVID-19 was determined by the following questions “Have you personally known a person that is COVID-19 positive?”, “Have you known any person who has died because of COVID-19?”. Participants were further inquired to report their infection status as either a confirmed case of COVID-19, had a contact with a positive COVID-19 case, suspected case with symptoms or none. 

### 2.2. Statistical Analysis

Analysis of the data collected was performed using Stata Statistical Software (Release 14, College Station, TX, USA: StataCorp LP, 2015.). Descriptive statistics included percentages for categorical variables, and means with standard deviations for continuous variables. Regrouping of some variables was made based on their distribution, such as: perceived status of health, perceived status of oral health, and importance of oral health. In the perceived status of general health and oral health variables, ‘fair’ and ‘poor’ groups were combined. The majority of the respondents rated the importance of oral health as ‘very important’, thus other categories were merged into ‘less important’. Pearson’s r correlation coefficient, analysis of variance (ANOVA), and independent t-test were used to detect differences in fear to seek dental care during COVID-19 outbreak across socio-demographic and other key variables. Multiple linear regressions were further employed to identify which variables could explain the impact of fear of COVID-19 on seeking dental care. The level of acceptable significance was set at *p* < 0.05.

## 3. Results

### 3.1. Sample Characteristics

The sample included 826 subjects (Table 1), of which 34.50% (*n* = 285) were men and 65.50% (*n* = 541) were women, with mean age of 38.84 ± 13.29 ranging from 18 to 70 years. Most of the participants were Saudi (93.95%), and higher percentage of the subjects were from Makkah and Eastern Provinces (31.96 and 30.75%, respectively). In terms of education level, about 55.81% obtained a bachelor’s degree, and 19.13% had a graduate degree. One third of the participants earn a monthly income of more than 20,000 Saudi Riyal, and 24.46% reported working in the medical field. 

### 3.2. Attitudes and Behaviors toward Health and Health Care

The attitudes and behaviors of participants toward health and health care is presented in (Table 2). Most of the participants perceived the status of their general health as good or excellent (48.55 and 45.16%, respectively). Nearly half of the sample population perceived their oral health status as good, and about 90% of the participants rated the importance of oral health as high. 

About forty-two percent of the sample reported visiting the dentist only for emergency, 66.46% did not have a regular dentist, and a high percentage of the sample reported not visiting the dentist within the last 6 months (53.87%). In addition, 62.11% of the participants were aware of having untreated dental conditions.

### 3.3. COVID-19 Experience

In terms of previous COVID-19 infection, around 98% reported no history of COVID-19 infection at the time of data collection, 29% knew COVID-19 cases, and 19.25% knew a person who died due to COVID-19 (Table 2). More than two thirds of the sample perceived their risk of getting a COVID-19 infection through visiting dental clinics as moderate to high risk.

### 3.4. Fear of COVID-19

The total score of Fear of Getting Sick was 8 ± 3.12, Fear that Others Get Sick was 8.93 ± 3.20, Fear of the Impact on Social Life was 22.36 ± 6.99, and Behaviors Related to Illness and Virus Fears was 31.32 ± 6.21.

### 3.5. Fear to Seek Dental Care

The level of fear to seek dental care varied across demographic characteristics (Table 1). Females had significantly higher fear to seek dental care than males, 2.33 ± 1.0 and 2.18 ± 1.08, respectively at *p*: 0.03. Participants 45 years and older had less fear to seek dental care compared to younger age groups (*p <* 0.001). There were no significant differences in fear to seek dental care during the pandemic across other demographic variables. 

Fear to seek dental care was shaped by individual’s health attitude (Table 2). Those who perceived their general health as fair to poor had significantly higher level of fear to seek dental care (2.69 ± 1.16, *p <* 0.001). Similarly, the participants who perceived their oral health as fair to poor had significantly higher level of fear to seek dental care (2.54 ± 1.02, *p <* 0.001). Reporting untreated dental conditions was also found to be significantly associated with high level of fear (2.40 ± 1.02, *p <* 0.001). Compared to who had a recent dental visit within the last 6 months, participants who failed to see a dentist within the last 6 months were more likely to experience fear to seek dental care (2.35 ±1.03, *p*: 0.02).

The perceived risk of COVID-19 infection in dental clinics was also significantly associated with fear to seek dental care, as the ones who graded their risk as high (2.86 ± 1.05) or moderate (2.19 ± 0.81) had significantly high fear to seek dental care.

Fear to seek dental care was further significantly correlated with each of Fears of Illness and Virus Evaluation subscales in positive direction: Fear that Others Get Sick (*r*: 0.36, *p* < 0.001), Fear of Getting Sick (*r*: 0.35, *p <* 0.001), Fear of the Impact on Social Life (*r*: 0.35, *p <* 0.001), and least correlation yet significant was with Behaviors Related to Illness and Virus Fears (*r*: 0.28, *p* < 0.001).

### 3.6. Predictors of Fear to Seek Dental Care

The predictors of fear to seek dental care were further evaluated in a multivariate environment to control for the effect of cofounders (Table 3). The analysis was conducted in stages where variables were entered into the models gradually. 

At first, the model was adjusted only for gender and age, and it did not show any statistically significant relationship. 

In the second model, gender, age, and the four subscales of the FIVE were fitted in the model. The age group of (35–44 years) showed a higher likelihood of being afraid to seek dental care when compared to the reference (β: 0.20, 95%Confidence Interval (CI): 0.01, 0.40, *p*: 0.04) when all other variables in the model were held constant. Fear of Getting Sick and Fear that Others Get Sick were significantly associated with higher fear to seek dental care (β: 0.04, 95%CI: 0.01, 0.07, *p <* 0.01) and (β: 0.06, 95%CI: 0.04, 0.09, *p <* 0.001), respectively. Fear of the Impact on Social Life and Behaviors Related to Illness and Virus Fears were also significantly associated with high levels of fear (β: 0.02, 95%CI: 0.01, 0.03, *p <* 0.001) and (β: 0.02, 95%CI: 0.01, 0.03, *p <* 0.01), respectively.

The third model was adjusted for all previously significant variables at bivariate analysis in Table 1 and Table 2. The age group of (35–44 years) kept its higher likelihood of experiencing fear to seek dental care (β: 0.19, 95%CI: 0.01, 0.38, *p*: 0.04). Fear that Others Get Sick was significantly associated with higher fear to seeking dental care (β: 0.04, 95%CI: 0.02, 0.07, *p <* 0.001). Fear of the Impact on Social Life (β: 0.02, 95%CI: 0.01, 0.03, *p <* 0.001) and Behaviors Related to Illness and Virus Fears (β: 0.02, 95%CI: 0.01, 0.03, *p <* 0.01) were significantly associated with fear. The participants who perceived moderate risk and high risk of COVID-19 infection in dental clinics had a significantly higher likelihood of fear (β: 0.51, 95%CI: 0.25, 0.78, *p <* 0.001) and (β: 1.02, 95%CI: 0.76, 1.29, *p <* 0.001), respectively. The participants who reported having untreated dental conditions had a higher likelihood of reporting fear (β: 0.25, 95%CI: 0.09, 0.39, *p <* 0.01) than those who reported no untreated conditions when all other variables were held constant in the model.

## 4. Discussion

The unpleasant feeling of fear is triggered by many personal and environmental factors. The recent pandemic has affected people’s daily life and their psychological stability worldwide. Healthcare is one of the highly affected sectors during the recent pandemic including dental care. Seeking healthcare is essential for maintaining good health. The present study aimed to measure fear to seek dental care among Saudi population when needed during COVID-19 pandemic. Our findings delineated that higher fear to seek dental care was significantly associated with higher fear of COVID-19 and perception of higher risk of infection in dental settings. Participants with untreated dental conditions had significantly higher likelihood of experiencing fear to seek dental care.

Population’s strong feeling of vulnerability to an infection and perceived germ aversion were associated with higher fear of COVID-19 and subsequent reluctance in seeking dental care during the pandemic in a previous study on a sample of the Spanish population [31]. Similarly, the results of this study reflect the significant association between fear to seek dental care and Fear that Others Get Sick (β: 0.06, 95%CI: 0.04, 0.09, *p <* 0.001), Fear of the Impact on Social Life (β: 0.02, 95%CI: 0.01, 0.03, *p <* 0.001), and Behaviors Related to Illness and Virus Fears (β: 0.02, 95%CI: 0.01, 0.03, *p <* 0.01). Such findings are predictable since the situation during the pandemic created an increased level of stress and uncertainty, especially when the disease transmission was not clearly understood early in the pandemic [13]. Fear to seek dental care during the pandemic was more related to population’s fear of hurting others rather than self, and to the impact of the disease on their social life including isolation from friends and family, and the failure to perform well in their jobs. 

COVID-19 can be transmitted through small airborne micro-droplets [32]. Dental teams are the most susceptible workers to COVID-19 infection due to the nature of their job that put them at higher risk for virus exposure through close physical proximity to others and through performing aerosol generating procedures [24,33]. In the present study, over 60% of the respondents perceived moderate to high risk of contracting infection through dental clinics. Moderate and high risk of contracting COVID-19 infection through dental clinics were strongly associated with fear to seek dental care (β: 0.51, 95%CI: 0.25, 0.78, *p <* 0.001) and (β: 1.02, 95%CI: 0.76, 1.29, *p <* 0.001), respectively. The finding is supported by previous investigations in which 30% of a Spanish sample [34], 80% of a Chinese sample [35], and 58% of a Saudi sample [28] reported their reluctance to visit the dentist because of the possibility of getting infected by COVID-19. Future intentions to reduce the reluctance to seek dental care should be directed toward people at higher risk of developing fear. This could be through integrating preventive oral care and measures in primary care, community-based preventive programs, and assuring patients by explaining the precautionary measures taken in dental clinics.

In the current study, fear to seek dental care was high among those who reported having untreated dental conditions (β: 0.25, 95%CI: 0.09, 0.39, *p <* 0.01). The association between oral health conditions as dental caries and periodontal diseases with psychological conditions as stress, anxiety, and depression is strongly documented in the literature [36]. It is also possible that delayed dental visits due to the pandemic could have resulted in a higher number of untreated dental conditions among this population. 

Factors such as stress, poor diet, tobacco use, poverty, and behavioral health issues are among common risk factors that affect health generally including oral health. Many of these factors were elevated during the pandemic. People at a higher risk of severe COVID-19 symptoms and death, such as elderly, low socioeconomic status, low literacy, and people with chronic diseases, such as diabetes and cardiovascular disease, are at higher risk of oral diseases. Therefore, this population are at a higher need for prevention of oral diseases. Adopting caries preventive approach and non-aerosol generating management of dental diseases would be crucial in addressing this issue [37]. 

The findings of the current study should be interpreted with cautions to its limitations. The non-probability convenient sampling technique using the application on smartphones could affect the generalizability of the study findings. Another shortcoming of this study is that a cause–effect relationship could not be established due to its cross-sectional nature. A prospective longitudinal study would be needed to establish the causality of some found relationships. The use of a self-report measure introduced the probability of recall bias and social desirability. In addition, the omittance of some items in FIVE would affect its total scores that may make future comparisons challenging. In this study, a quiet large sample size was recruited as well as a validated multidimensional questionnaire (FIVE; Ehrenreich-May) was used which could display the strengths of the study. 

Fear can fluctuate over time leading to changes in health behaviors as seeking healthcare. Thus, it is recommended to assess the influence of the pandemic on fear to seek dental care in relation to time using longitudinal study design. It is also suggested that researchers in the future address the effect of different preventive measures in reducing dental conditions during pandemics, especially among fearful population.

## 5. Conclusions

Within the limitations of the present study, COVID-19 related fear was associated with fear to seek dental care. Those who perceived higher risk of COVID-19 infection in dental clinics and experienced untreated dental conditions were associated with higher fear to seek dental services during the pandemic.

## Figures and Tables

**Table 1:** Differences in fear to seek dental care by sociodemographic characteristics presented by means and standard deviations.

	Categories	Frequency (%)	Fear to Seek Dental Care Mean (SD)	*p*-Value
Gender	Male	285 (34.50)	2.18 (1.08)	0.03
	Female	541(65.50)	2.33 (1.00) *	
Age (years)	18–24	158 (19.13)	2.29 (0.96)	0.00
	25–34	184 (22.28)	2.38 (0.99)	
	35–44	198 (23.97)	2.42 (1.05)	
	45–54	162 (19.61)	2.06 (1.09) *	
	55 and older	124 (15.01)	2.19 (1.01)	
Citizenship	Saudi citizens	776 (93.95)	2.29 (1.03)	0.52
	Non-Saudi residents	50 (6.05)	2.19 (0.99)	
Region	Riyadh	201 (24.33)	2.31 (0.98)	0.49
	Makkah	264 (31.96)	2.34 (1.04)	
	Eastern	254 (30.75)	2.20 (1.05)	
	Other	107 (12.95)	2.28 (1.05)	
Education	High school or below	127 (15.38)	2.22 (0.99)	0.07
	Diploma degree	80 (9.69)	2.03 (1.01)	
	Bachelor’s degree	461 (55.81)	2.34 (1.04)	
	Graduate degree	158 (19.13)	2.28 (1.04)	
Monthly income(SAR)	Less than 5000	74 (8.96)	2.19 (0.91)	0.12
	5000–10,000	150 (18.16)	2.37 (1.10)	
	10,001–15,000	147 (17.79)	2.25 (1.03)	
	15,001–20,000	174 (21.07)	2.13 (0.94)	
	More than 20,000	281 (34.02)	2.36 (1.07)	
Working in medical field	Yes	202 (24.46)	2.17 (0.98)	0.09
	No	624 (75.54)	2.31 (1.05)	

* Significantly different at *p <* 0.05, SD = Standard Deviation, SAR = Saudi Arabian Riyal.

**Table 2:** Comparison of fear to seek dental care during COVID-19 outbreak by key variables presented by means and standard deviations.

	Categories	Frequency (%)	Fear to Seek Dental Care Mean (SD)	*p*-Value
Perceived status of general health	Excellent	373 (45.16)	2.18 (1.03)	0.00
	Good	401 (48.55)	2.32 (1.00)	
	Fair to poor	52 (6.29)	2.69 (1.16) *	
Perceived status of oral health	Excellent	208 (25.18)	2.16 (1.08)	0.00
	Good	411 (49.76)	2.21 (0.98)	
	Fair to poor	207 (25.06)	2.54 (1.02) *	
Importance of oral health	Very important	739 (89.47)	2.29 (1.03)	0.13
	Less important	87 (10.53)	2.17 (1.06)	
Frequency of dental visits	More than once a year	200 (24.21)	2.31 (1.09)	0.68
	About once a year	264 (31.96)	2.31 (1.03)	
	Only for emergency	345 (41.77)	2.26 (0.99)	
	Never	17 (2.06)	2.03 (0.95)	
Last dental visit within 6 months	Yes	381 (46.13)	2.19 (1.03)	0.02
	No	445 (53.87)	2.35 (1.03) *	
Untreated dental conditions	Yes	513 (62.11)	2.40 (1.02) *	0.00
	No	235 (28.45)	2.05 (1.02)	
	I don’t know	78 (9.44)	2.17 (1.01)	
Regular dentist	Yes	277 (33.54)	2.19 (0.99)	0.08
	No	549 (66.46)	2.32 (1.05)	
COVID-19 infection status	Positive	2 (0.24)	1.00 (0)	0.06
	Contact with a positive case	13 (1.57)	2.85 (0.80)	
	Suspected case with symptoms	6 (0.73)	2.50 (0.95)	
	None	805 (97.46)	2.27 (1.03)	
Know COVID-19 cases	Yes	236 (28.57)	2.30 (1.06)	0.68
	No	590 (71.43)	2.27 (1.02)	
Know COVID-19 deaths	Yes	159 (19.25)	2.29 (1.03)	0.79
	No	667 (80.75)	2.28 (1.03)	
Perceived risk of COVID-19 infection in dental clinics	No risk	48 (5.81)	1.59 (0.86)	0.00
	Low risk	203 (24.58)	1.69 (0.81)	
	Moderate risk	272 (32.93)	2.19 (0.81) *	
	High risk	303 (36.68)	2.86 (1.05) *	

* Significantly different at *p <* 0.05, SD = Standard Deviation.

**Table 3:** Factors associated with higher fear to seek dental care during COVID-19 outbreak in Saudi Arabia (n= 826).

		Model 1			Model 2			Model 3		
	Categories	β Coefficient	*p*-Value	95% CI	β Coefficient	*p*-Value	95% CI	β Coefficient	*p*-Value	95% CI
Gender	Male	Reference			Reference			Reference		
	Female	0.12	0.12	−0.03, 0.27	0.05	0.48	−0.09, 0.18	0.02	0.75	−0.10, 0.14
Age (years)	18–24	Reference			Reference			Reference		
	25–34	0.08	0.48	−0.14, 0.29	0.05	0.64	−0.15, 0.25	0.01	0.89	−0.17, 0.19
	35–44	0.12	0.27	−0.09, 0.33	0.20	0.04	0.01, 0.40	0.19	0.04	0.01, 0.38
	45–54	−0.22	0.06	−0.44, 0.01	−0.00	0.98	−0.21, 0.21	0.05	0.59	−0.14, 0.25
	55-older	−0.09	0.47	−0.33, 0.15	0.03	0.83	−0.20, 0.25	0.14	0.19	−0.07, 0.36
FIVE Subscales	Fear of Getting Sick				0.04	0.00	0.01, 0.07	0.02	0.19	−0.01, 0.04
	Fear that Others Get Sick				0.06	0.00	0.04, 0.09	0.04	0.00	0.02, 0.07
	Fear of the Impact on Social Life				0.02	0.00	0.01, 0.03	0.02	0.00	0.01, 0.03
	Behaviors Related to Illness and Virus Fears				0.02	0.00	0.01, 0.03	0.02	0.00	0.01, 0.03
Risk of COVID-19 infection in dental clinics	No risk							Reference		
	Low							0.10	0.44	−0.16, 0.37
	Moderate							0.51	0.00	0.25, 0.78
	High							1.02	0.00	0.76, 1.29
Perceived status of general health	Excellent							Reference		
	Good							−0.01	0.89	−0.14, 0.12
	Fair to poor							0.15	0.25	−0.11, 0.42
Perceived status of oral health	Excellent							Reference		
	Good							0.03	0.72	−0.13, 0.19
	Fair to poor							0.15	0.15	−0.05, 0.35
Last dental visit within 6 months	Yes							Reference		
	No							0.07	0.24	−0.05, 0.19
Untreated dental conditions	No							Reference		
	Yes							0.25	0.00	0.09, 0.39
	Don’t know							0.15	0.18	−0.07, 0.38

## Data Availability

The data presented in this study are available on request from the corresponding author.
